# The CD8 and CD4 T-Cell Response against Kaposi's Sarcoma-Associated Herpesvirus Is Skewed Towards Early and Late Lytic Antigens

**DOI:** 10.1371/journal.pone.0005890

**Published:** 2009-06-17

**Authors:** Rebecca C. Robey, Dimitrios Lagos, Fiona Gratrix, Stephen Henderson, Nick C. Matthews, Richard J. Vart, Mark Bower, Chris Boshoff, Frances M. Gotch

**Affiliations:** 1 Department of Immunology, Imperial College London, London, United Kingdom; 2 UCL Cancer Institute, University College London, London, United Kingdom; University of California San Francisco, United States of America

## Abstract

Kaposi's sarcoma-associated herpesvirus (KSHV) is causally related to Kaposi's sarcoma (KS), the most common malignancy in untreated individuals with HIV/AIDS. The adaptive T-cell immune response against KSHV has not been fully characterized. To achieve a better understanding of the antigenic repertoire of the CD8 and CD4 T-cell responses against KSHV, we constructed a library of lentiviral expression vectors each coding for one of 31 individual KSHV open reading frames (ORFs). We used these to transduce monocyte-derived dendritic cells (moDCs) isolated from 14 KSHV-seropositive (12 HIV-positive) and 7 KSHV-seronegative (4 HIV-positive) individuals. moDCs were transduced with up to 3 KSHV ORFs simultaneously (ORFs grouped according to their expression during the viral life cycle). Transduced moDCs naturally process the KSHV genes and present the resulting antigens in the context of MHC class I and II. Transduced moDCs were cultured with purified autologous T cells and the CD8 and CD4 T-cell proliferative responses to each KSHV ORF (or group) was assessed using a CFSE dye-based assay. Two pools of early lytic KSHV genes ([ORF8/ORF49/ORF61] and [ORF59/ORF65/K4.1]) were frequently-recognized targets of both CD8 and CD4 T cells from KSHV seropositive individuals. One pool of late lytic KSHV genes ([ORF28/ORF36/ORF37]) was a frequently-recognized CD8 target and another pool of late genes ([ORF33/K1/K8.1]) was a frequently-recognized CD4 target. We report that both the CD8 and CD4 T-cell responses against KSHV are skewed towards genes expressed in the early and late phases of the viral lytic cycle, and identify some previously unknown targets of these responses. This knowledge will be important to future immunological investigations into KSHV and may eventually lead to the development of better immunotherapies for KSHV-related diseases.

## Introduction

Kaposi's sarcoma-associated herpesvirus (KSHV; also known as human herpesvirus 8 [HHV-8]) is the etiological agent of Kaposi's sarcoma (KS), the most frequently-arising malignancy in untreated individuals with HIV/AIDS [1] and consequently one of the most common cancers in Sub-Saharan Africa [2]. KSHV is also involved in the pathogenesis of at least two lymphoproliferative disorders, primary effusion lymphoma (PEL) [3] and multicentric Castleman's disease (MCD) [4].

In immunocompetent individuals KSHV can establish life-long, asymptomatic infection. However, when immune control declines (for example, during AIDS) KSHV-related tumors may develop. KS is over 100 times more common in HIV-infected individuals than in immunocompetent individuals [1]. Moreover, spontaneous tumor regression is seen in individuals with KS when immunosuppression is reversed through highly-active antiretroviral therapy (HAART) [5], and this has been shown to correlate with a quantitative increase in KSHV-specific CD8 T-cell responses [6]–[8]. Likewise, KSHV-specific CD8 responses have been found to be of higher frequency and with greater diversity in their antigenic repertoire in asymptomatic carriers of KSHV compared to individuals with KS [9], [10]. Longitudinal studies of two individuals with KS found a correlation between reduced levels of KSHV-specific CD8 T cells and recurrence of active KS [10], [11]. Together, these findings infer that KS oncogenesis is associated with loss of T cell-mediated control of KSHV-infected cells.

T-cell responses have been detected against several lytic and latent KSHV proteins [8]–[10], [12]–[19]. Some of these responses have been shown to be functionally cytotoxic *in vitro* [15], [18] and there is evidence that they exert evolutionary pressure on the virus *in vivo* [17]. A few KSHV-specific T-cell epitopes have been identified [8]–[10],[12]–[14], [16], [17], [20] but these are almost exclusively CD8 epitopes and they elicit weak responses compared to epitopes from other viruses such as HIV-1 and EBV [12], [13], indicating that there may be immunodominant epitopes yet to be determined. There has been a limited number of investigations into the CD4 T-cell response against KSHV: one group reported the identification of two CD4 T-cell epitopes in K12 and K15 in one individual with AIDS-KS [9]. Thus, neither the breadth of the antigenic repertoire of the KSHV-specific T-cell immune response, nor its immunodominant targets, are fully understood.

In previous studies, a necessary limiting factor has been the size and complexity of the KSHV genome. Each study has been confined to analysis of a handful of genes, selected according to their homology with immunogenic genes from other γ-herpesviruses [10]; their expression profile [14], [16]; or evidence of sequence variation arising from immunological pressure [17]. Epitope identification has been performed using overlapping peptides for smaller genes [8], [9], [12], [17] or predictive algorithms for peptide HLA-binding affinity for larger genes [9], [13], [14], [16], [20].

This study further investigates the immunogenic profile of KSHV using monocyte-derived dendritic cells (moDCs) lentivirally-transduced to express 31 different KSHV open reading frames (ORFs) to perform a large-scale screen for immunogenicity. Lentiviral vectors efficiently deliver foreign genetic material into non-dividing cells such as moDCs [21] and integrate into the cellular genome resulting in sustained transgene expression [21]–[23]. Using overlapping peptides for this number of gene products would be impractical, and even the use of epitope prediction software (Immune Epitope Database; www.immuneepitope.org.uk) yields over 1000 potential nine-mer epitopes (IC50 value less than 5000 nM) from these 31 genes for HLA-A*0201 alone (unpublished data). Lentiviral delivery of an antigenic gene into moDCs allows the moDCs to present the naturally-occurring optimal CD8 and CD4 epitopes, thereby avoiding the limitations associated with the use of pre-determined peptides. No prior knowledge of the optimal peptide or HLA-restriction is required [24], [25]. Lentiviral-transduced moDCs have been used to stimulate both primary and recall antigen-specific T-cell responses *in vitro* [23], [26]–[29]. Such moDCs efficiently process and present both MHC class I- and II-restricted epitopes and thereby prime both CD8+ and CD4+ T cell responses [30], [31]. One group has reported the use of lentiviral-transduced moDCs expressing a melanoma-specific antigenic protein to isolate an anti-melanoma CD8+ T cell that recognized a previously unknown peptide [32].

Using this system, we report that both the CD8 and CD4 T cell responses against KSHV are directed predominantly towards genes expressed in the early and late lytic phases of the viral life cycle, and we have identified novel immunogenic targets for future investigations into host immune control of KSHV infection.

## Results

### moDCs can be lentivirally transduced to stably express GFP or KSHV ORFs

**Figure 1:**
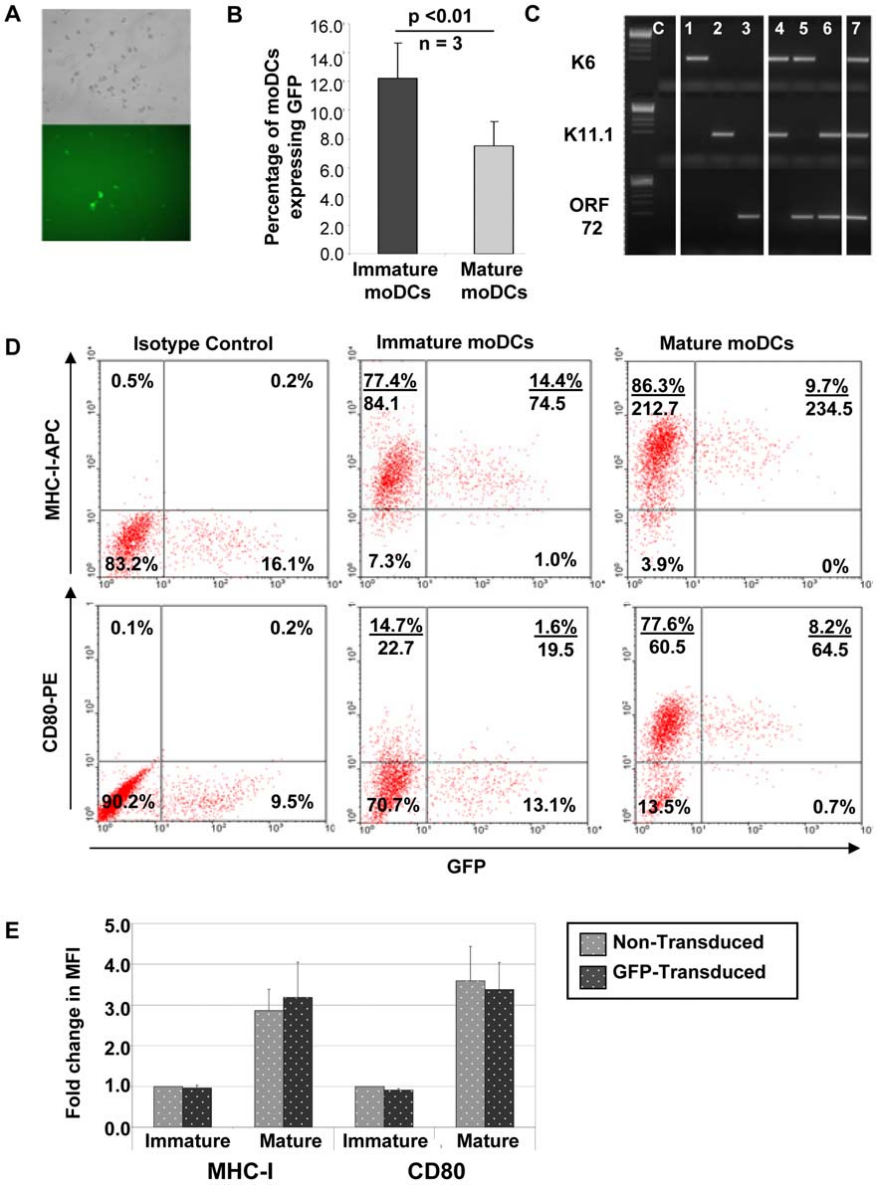
Lentiviral transduction of moDCs. (a) Immature moDCs transduced with GFP-encoding lentivirus, day 6 post-transduction. Top panel shows phase contrast, bottom panel shows GFP fluorescence. (b) Proportion of GFP-positive moDCs at day 6 post-transduction. Means and standard deviations are shown (n = 3). (c) Expression of KSHV ORFs K6 (top band), K11.1 (middle band) and ORF 72 (bottom band) by moDCs on day 6 post-transduction with one (lanes 1 to 3); two (lanes 4 to 6); or three (lane 7) KSHV-gene-encoding lentiviruses. (d) Examples of flow cytometry dot plots showing MHC-I (top panels) and CD80 (bottom panels) surface expression by GFP-transduced and non-transduced moDCs before and after maturation. Numbers in each quadrant indicate the percentage of cells in that quadrant (top) and the MHC-I or CD80 mean fluorescence intensity (MFI) of the cells in that quadrant (bottom). (e) Fold change in MHC-I and CD80 surface expression by non-transduced and GFP-transduced moDCs before and after maturation. Mean and standard deviations from three experiments are shown. In each experiment, MFIs were normalized to non-transduced, immature moDCs.

The green fluorescence protein (GFP)-encoding lentivirus pCSGW (the vector from which our pSIN vector was derived) was used to demonstrate that moDCs can be successfully transduced using our lentiviral vector and to investigate the kinetics of transgene expression and the optimal multiplicity of infection (MOI) for our vector. Transduction with pCSGW resulted in GFP expression by moDCs (Figure 1a). A time-course experiment revealed that GFP transgene expression increased steadily over several days (data not shown), as has been reported by other groups [21], [23]. A six-day period was selected as a suitable length of time for culture of transduced moDCs, representing a balance between obtaining good transgene expression and optimal moDC viability. After six days, a transduction efficiency of 12.2±2.5% (mean±s.d.) GFP-positive immature moDCs was observed (n = 3) (Figure 1b). Interestingly, a downregulation of GFP transgene expression was observed after maturation of moDCs (7.5±1.7% GFP-positive cells). A titration experiment was performed to assess the optimal multiplicity of infection (MOI) for the vector (data not shown) and a target MOI of between 3 and 8 for each transduction was selected. With our GFP construct this achieved good transgene expression (between 11 and 15% GFP-positive immature moDCs, data not shown), with no notable improvement if the MOI was increased above 8. Furthermore, whilst there is a consensus that lentiviral transduction of moDCs at MOIs of less than 10 does not affect moDC viability, immunophenotype or antigen-presenting function [23], [30], [33], [34], the evidence regarding transduction with higher MOIs is less clear [35].

**Figure 2:**
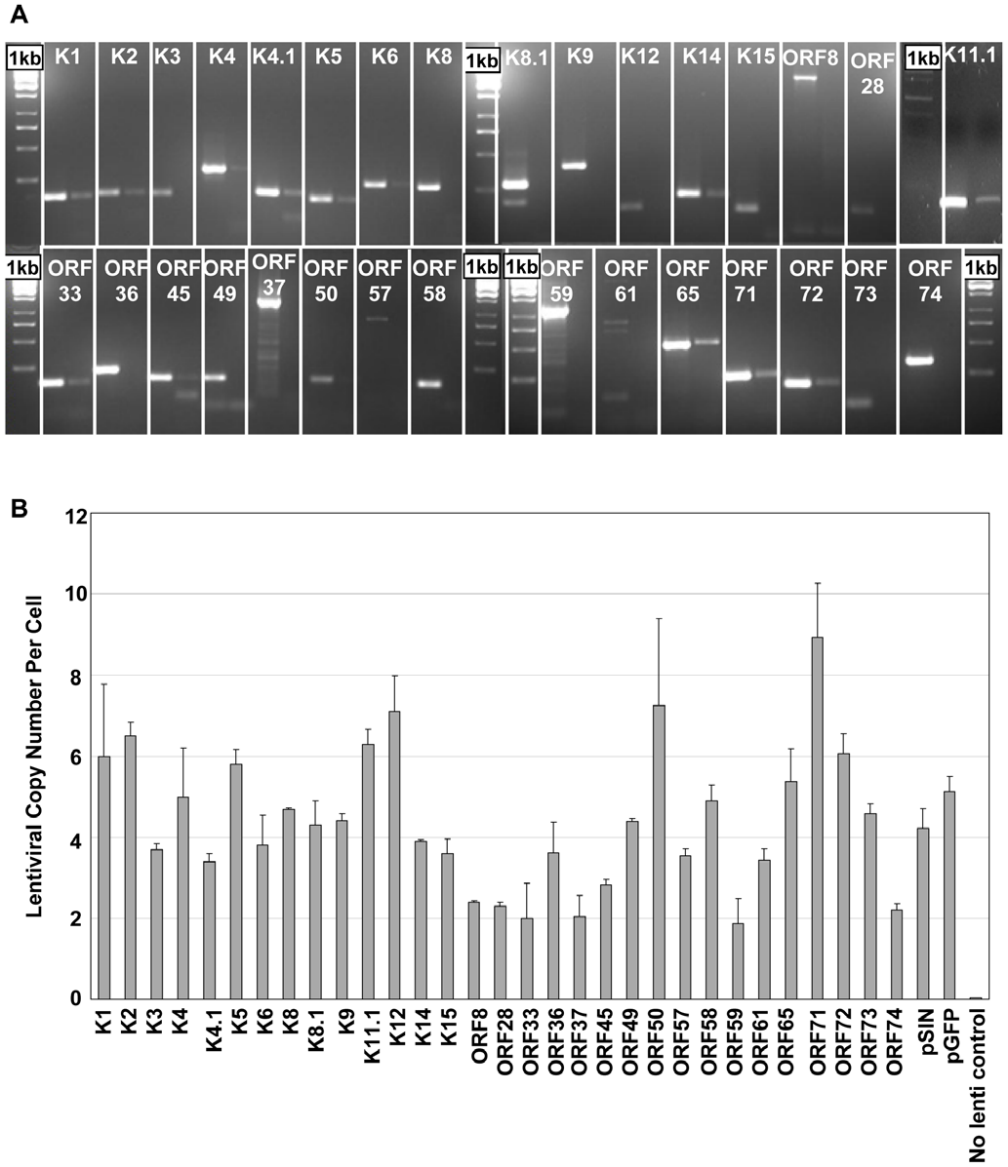
Expression and multiplicity of infection in moDCs of each of the KSHV-gene-encoding lentiviruses. (a) KSHV ORF expression by mature moDCs 6 days after transduction with KSHV-gene-encoding lentiviruses. For each ORF, the left-hand lane shows the reverse transcription (RT)-PCR product and the right-hand lane shows the no reverse transcriptase control. (b) Multiplicity of Infection (MOI) of each of the KSHV-gene-encoding lentiviral preparations in one experiment. Average lentiviral copy number per cell and standard deviations of duplicates are shown.

We used RT-PCR to ensure that all KSHV ORFs were expressed by moDCs after lentiviral transduction (Figure 2a) and quantitative PCR to titre all KSHV-gene-encoding lentivirus preparations (Figure 2b). The volume of each lentivirus used in all subsequent experiments was then adjusted to achieve a uniform MOI of between 3.4 and 7.2 lentiviral copies per cell for all preparations (median 4.5; interquartile range 3.9 to 5.4; mean 4.75). This range was selected based on the results from experiments with our GFP construct discussed above.

As the KSHV lentiviral library consists of 31 KSHV ORFs we decided initially to perform our immunogenic screen with moDCs transduced with up to three different KSHV ORFs simultaneously, in order to make the experiments more manageable and to make the best use of clinical samples. KSHV ORFs were grouped according to their expression profile to determine whether latent, immediate-early, early or late lytic gene products elicit the strongest T-cell responses. The classification of KSHV ORFs was based on their expression in PEL cells [36], [37]. We performed a multiple transduction experiment and used RT-PCR to demonstrate that moDCs can be transduced to express one, two or three KSHV ORFs simultaneously (Figure 1c).

### Lentiviral transduction does not affect moDC's antigen-presenting surface phenotype or moDC maturation

We used GFP-transduced moDCs to examine MHC-I and CD80 surface expression by non-transduced and lentiviral-transduced moDCs. There was no difference between the MHC-I or CD80 mean fluorescence intensities (MFIs) of non-transduced or transduced immature moDCs, and these markers were equally upregulated by non-transduced and transduced moDCs after exposure to maturation stimuli (Figure 1d and 1e). This indicates that lentiviral transduction does not affect the antigen-presenting surface phenotype of moDCs or moDC maturation.

### T-cell proliferation responses to moDCs transduced to express KSHV genes

moDCs were isolated from 14 KSHV seropositive (12 HIV+) individuals and 7 KSHV-seronegative (4 HIV+) individuals. Characteristics of all study participants are shown in Table 1.

**Table 1:** Summary of study participant characteristics.

	Number	Male/Female	Mean Age (Range)	KSHV status	HIV status	Mean CD4 Count (Range)
HIV-/KSHV- Healthy Controls	3	3/0	37 (30 to 45)	Seronegative	Negative	ND
HIV+/KSHV- Controls	4	3/1	48 (32 to 70)	Seronegative	Positive; On HAART; HIV viral load suppressed	345 (174 to 652)
HIV+/KSHV+; history of KSHV related neoplasia	11	11/0	47 (36 to 74)	Seropositive*; KSHV-related disease in remission	Positive; On HAART; HIV viral load suppressed	389 (171 to 785)
HIV+/KSHV+; asymptomatic carriers of KSHV	1	1/0	56	Seropositive; Asymptomatic	Positive; On HAART; HIV viral load suppressed	373
HIV-/KSHV+; history of KSHV related neoplasia	2	2/0	37, 41	Seropositive; Active KS, regressing	Negative	550, ND

* Except for 2 patients (P3 and P5) for whom plasma were unavailable for serological testing; ND = not determined.

**Figure 3:**
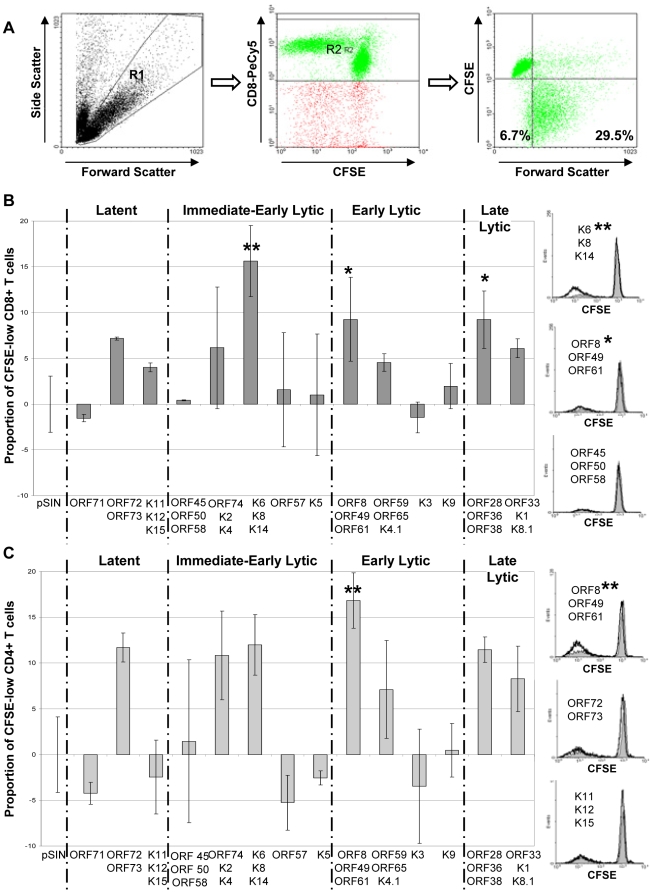
T-cell proliferation responses to transduced moDCs. (a) Gating strategy for T-cell proliferation response assays. Gates are set on the live T lymphocyte population (left panel) and CD8+ cells (middle panel), and the proportion of proliferating cells is taken as the percentage of CFSE-low cells in the bottom two quadrants (right panel). For CD4 responses, R2 is readjusted over the CD8-low cells. (b) Example of CD8 responses to moDCs transduced with each KSHV gene or group of genes from one study participant (P7; HIV+, KSHV+, history of KSHV-related disease, in remission). Bar graphs show the proportion of CFSE-low cells in response to each gene or group of genes (with the background response to moDCs transduced with the empty pSIN vector subtracted). Mean of triplicates and standard deviations are shown. Two stars indicate a response that was considered to be positive; one star indicates a response that was considered to be borderline positive according to the criteria described in Materials and Methods. Flow cytometry histograms show examples of strong (top), medium (middle) and negative (bottom) CD8 responses to different KSHV antigens from the same study participant (P7). In all histograms, grey shading represents CD8 T cells cultured with moDCs transduced with the empty pSIN vector and black lines represent CD8 T cells cultured with moDCs transduced with different KSHV antigens. (c) Same as for (b), but showing CD4 responses from the same study participant (P7).

moDCs were transduced to express up to three KSHV ORFs (grouped according to their expression profile) and then cultured with autologous CFSE-stained T cells. KSHV ORFs known to affect MHC-I expression (K3 and K5 [38]; and K9 and ORF 71 [39]) were used to singly transduce moDCs, since these genes' function may affect T-cell priming by moDCs thus skewing the results. After six days, T cells were harvested and flow cytometry was used to assess the CD8-positive cytotoxic lymphocyte (CTL) response and the CD8-negative (CD4) helper T-cell response to each KSHV ORF or pool, as measured by the proportion of CFSE-low proliferating cells (Figure 3a). An example of CD8 and CD4 responses by one HIV-positive, KSHV-seropositive individual (P7) are shown in Figures 3b and 3c, respectively. We used strict criteria to designate positive and borderline positive responses (see Materials and Methods) as we wished to ensure that no false positives were recorded as a result of the slight variation we observed in individuals' background response to moDCs transduced with the empty vector alone. This may mean that some weak T-cell responses were not identified in some or all KSHV-seropositive individuals. However, it allows us to observe patterns of immunodominant T-cell responses against KSHV.

**Figure 4:**
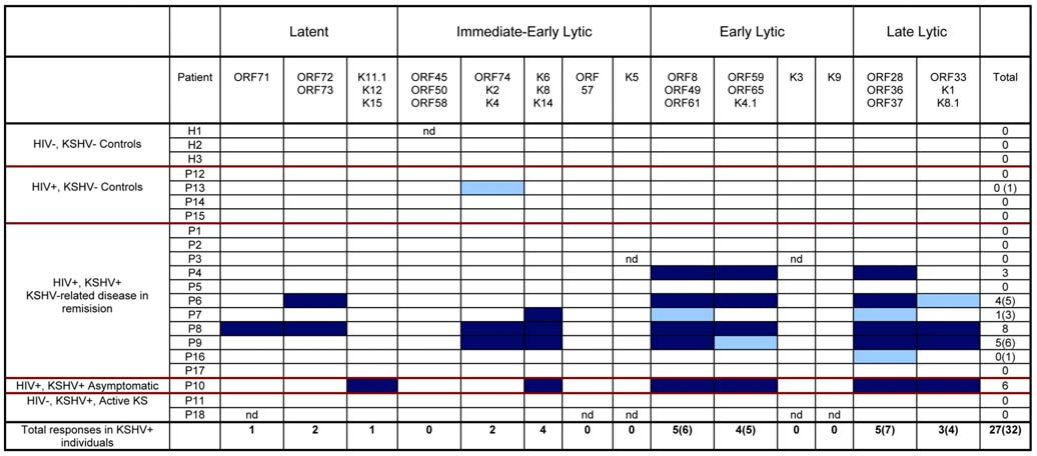
Summary of CD8 responses to KSHV ORFs by all study participants. Dark blue boxes represent positive responses; light blue boxes represent borderline responses; unfilled boxes represent no response; nd indicates not done due to insufficient numbers of available PBMCs.

**Figure 5:**
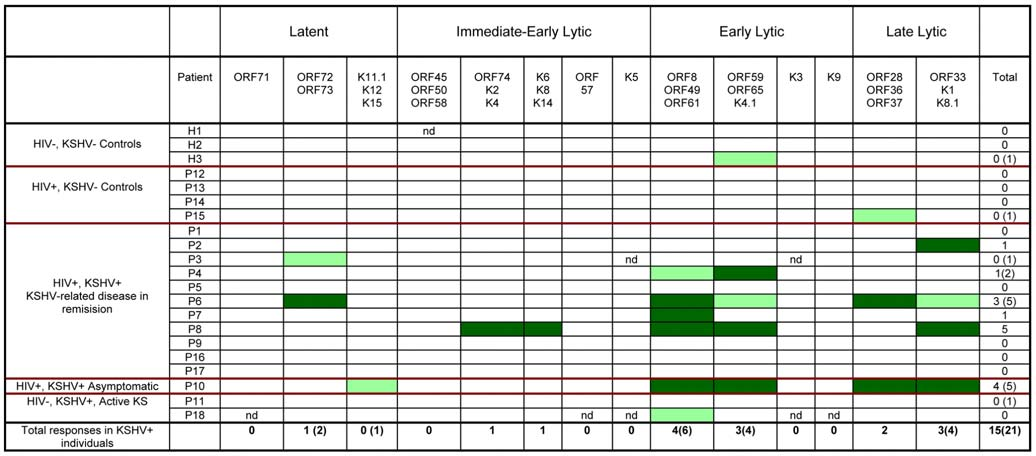
Summary of CD4 responses to KSHV ORFs by all study participants. Dark green boxes represent positive responses; light green boxes represent borderline responses; unfilled boxes represent no response; nd indicates not done due to insufficient numbers of available PBMCs.

CD8 and CD4 responses by all participants to all KSHV ORFs or pools are summarized in Figures 4 and 5 respectively. A single borderline CD8 response and two borderline CD4 responses were observed in KSHV seronegative individuals compared to 32 CD8 responses and 21 CD4 responses by KSHV seropositive individuals. All KSHV seronegative individuals gave strong positive responses to moDCs transduced to express an adenovirus encoding the immunodominant CMV gene phosphoprotein 65. This gives us confidence that the responses measured are KSHV-specific.

One of the two KSHV+, HIV- participants who had active classic KS at the time of venesection (P18) did not respond to any of the KSHV ORFs (CD8 or CD4) and the other (P11) gave only one borderline CD4 response. The KSHV+, HIV+ asymptomatic carrier of KSHV (P10) gave six strong positive CD8 responses; and one borderline and four strong CD4 responses to different KSHV ORFs. Of the 11 KSHV+, HIV+ individuals with a history of KSHV-related disease, six (P1, P2, P3, P5, P16 and P17) gave one or no responses (total of CD8 and CD4), and were classified as poor responders. There was no notable difference in the ages, CD4 counts or years in remission from KSHV-related disease between the poor responders and good responders (>1 response). However, interestingly, one poor responder (P1) suffered a relapse of KS within a year of venesection for this experiment. Three other poor responders (P2, P5 and P17) had received no cancer-specific chemotherapy treatment in addition to their antiretroviral therapy, whereas all good responders received HAART in combination with cancer chemotherapy. All poor responders had a history of KS alone, except for P3, who had been treated for PEL. Of the good responders, three (P6, P8 and P9) had a history of KS alone and two (P4 and P7) had a history of KS and MCD. These study participant characteristics are summarized in Table 2.

**Table 2:** Characteristics of HIV+ individuals who are in remission from KSHV-related disease.

	Patient	Age	CD4 Count	KSHV-related disease history	Treatment for KSHV-related disease	Approximate years between active disease and venesection
**Poor Responders**	P1	46	785	KS	HAART+chemotherapy+radiotherapy	<1, since relapsed
	P2	74	183	KS	HAART only	2
	P3	46	565	PEL	HAART+chemotherapy	3.5
	P5	39	406	KS	HAART only	<1
	P16	51	171	KS	HAART+chemotherapy	5
	P17	40	308	KS	HAART only	<1
	**Mean**	**49**	**403**			2
**Good responders**	P4	36	645	KS and MCD	HAART+chemotherapy	<1
	P6	38	496	KS	HAART+chemotherapy+radiotherapy	8.5
	P7	53	281	KS and MCD	HAART+chemotherapy	1.5
	P8	49	226	KS	HAART+chemotherapy	3.5
	P9	49	209	KS	HAART+chemotherapy	<1
	**Mean**	**45**	**371**			3

### Targets of the KSHV-specific CD8 CTL T-cell response

CD8 responses in all responsive KSHV+ individuals showed a bias towards early and late lytic gene products. Out of a total of 32 CD8 responses observed, 4 were directed against latent ORFs; 6 against immediate-early lytic ORFs; 11 against early lytic ORFs; and 11 against late lytic ORFs. Two pools of early lytic ORFs were frequently recognized CD8 targets: [ORF8/ORF49/ORF61] was recognized by 6 individuals and [ORF59/ORF65/K4.1] was recognized by 5 individuals. One pool of late lytic ORFs – [ORF28/ORF36/ORF37] – was recognized by 7 individuals, and was of particular interest to us as none of these gene products have been previously investigated for immunogenicity. For the same reason, a pool of immediate-early ORFs – [K6/K8/K14] – that was recognized by 4 individuals is also of interest.

### Targets of the KSHV-specific CD4+ T-cell response

CD4 responses across all individuals also showed a bias towards early and late lytic gene products. Out of a total of 21 CD4 responses observed, 3 were directed against latent ORFs; 2 against immediate-early lytic ORFs; 10 against early lytic ORFs; and 6 against late lytic ORFs. The most frequently recognized CD4 targets were the pool comprised of [ORF8/ORF49/ORF61], which was recognized by 6 individuals; and the pools [ORF59/ORF65/K4.1] and [ORF33/K1/K8.1], which were each recognized by 4 individuals.

### HLA Types of Good Responders

HLA typing was performed for all individuals classified as good responders. As might be expected of an ethnically diverse cohort of individuals from a central London clinic, the HLA types of these individuals were mixed and are shown in Table 3.

**Table 3:** HLA types of good responders.

	Participant	HLA Types					
		A	B	Cw	DRB1	DRB3 DRB4 DRB5	DQB1
**HIV+, KSHV-related neoplasia in remission**	**P4**	2, 29	7, 44	7, 16	7, 15	51, 53	2, 6
	**P6**	24, 29	51, 65	8, 15	4, 13	52, 53	6, 8
	**P7**	2, 3	7, 35	7, 4	1, 15	51	5, 6
	**P8**	2, 24	13, 44	6, 5	4, 15	51,53	6, 7
	**P9**	nd	nd	6,16	7,11	nd	nd
**HIV+, Asymptomatic**	**P10**	3	7, 62	7, 10	4, 15	51,53	6, 8

nd = not determined.

## Discussion

In this study, we have used lentiviral-transduced moDCs to express a panel of KSHV ORFs to investigate the adaptive CD8 and CD4 T-cell responses against KSHV. This approach has enabled us to perform a broad investigation into the KSHV-specific T-cell response, and has enabled us to identify antigenic hotspots within the KSHV genome.

Lentiviral transduction of moDCs with the conditions used did not affect the moDCs immunophenotype or maturation, as determined by expression of MHC-I and CD80. We showed that moDCs can be transduced with up to three different KSHV-gene-encoding lentiviruses simultaneously, resulting in expression of all three transgenes. Interestingly, we found that after maturation of moDCs, GFP expression was slightly down-regulated. Other groups have reported that mature moDCs are harder to transduce than immature moDCs [21], [33], but to the best of our knowledge there has been no previous documentation of decreased transgene expression when maturation is induced after transduction. This down-regulation may be the result of the terminal differentiation of mature moDCs and the concurrent changes in cellular gene expression and promoter activity. Importantly, even after maturation we achieved an average of 7.5% GFP-positive moDCs, a level comparable to those reported by other groups using a similar protocol [21], [23], [26], [28], [33].

Although our study is too small to draw definite conclusions regarding the differences in phenotypes of KSHV seropositive individuals who made several responses to KSHV antigens and those who did not, we show that individuals with active KS made few responses and an asymptomatic KSHV carrier made several responses, as has been previously described [9], [10]. We found that all individuals who had received HAART alone as treatment for their KS were poor responders, whereas all good responders had received HAART in combination with cytotoxic therapy. This concurs with studies that have found better reconstitution of KSHV-specific responses in individuals receiving HAART and cancer chemotherapy compared with those receiving HAART alone [6]. We also found that individuals with a history of MCD were good responders, in line with a previous report of individuals with active MCD who made responses to KSHV antigens comparable to those seen in asymptomatic carriers of KSHV [40].

In a cohort of 14 KSHV seropositive individuals, we saw a distinct skewing of both CD8 and CD4 responses towards early and late lytic gene products. This was an unexpected result, as it appears to contrast with observations of the T-cell response against Epstein-Barr virus (EBV; also known as HHV-4, the most closely related human γ-herpesvirus to KSHV), which preferentially targets immediate-early EBV gene products [41]–[44]. It has been suggested that immediate-early gene products are frequent targets of herpesvirus-specific T-cell responses as this enables activation of the host immune response before the expression of viral genes involved in immune evasion strategies. However, it has recently been reported that the CD8 T-cell response against the murine γ-herpesvirus 68 (MHV-68) is also directed against early and late lytic gene products [45]. A broad repertoire of epitopes derived from late lytic structural proteins and early lytic proteins that are involved in DNA replication were identified in MHV-68, but no epitopes derived from immediate-early proteins were found. Likewise, 86% of CD8 and 90% of CD4 T-cell responses against human cytomegalovirus (HCMV; a β-herpesvirus) target early and late lytic gene products [46]. Thus, protein abundance and, to a lesser degree, function may significantly affect the immunogenicity of the gene product. We further speculate that immunodominance of early and late lytic KSHV antigens may be an important factor in establishing homeostasis between host and virus. The activation of viral immune evasion strategies such as the down regulation of MHC molecules before the expression of immunodominant antigens may result in a blunting of the immune response, enabling the virus to avoid elimination and to establish controlled chronic infection.

We found that the three gene pools most frequently-recognized by CD8 T cells from KSHV+ individuals were [ORF8/ORF49/ORF61]; [ORF59/ORF65/K4.1]; and [ORF28/ORF36/ORF37]. ORF8 codes for glycoprotein B, which contains a well-described CD8 epitope, aa492–500 (LMWYELSKI) [9], [10], [20]. ORF61 codes for a large ribonucleotide reductase essential for DNA synthesis and also contains a previously documented CD8 epitope, aa505–513 (GLADVFAEL) [10]. ORF65 codes for the minor capsid protein which has also been identified as a target of the KSHV-specific CD8 T-cell response [19], and contains one identified CD8 epitope (aa35–43; NMSQAEYLV). However, none of the gene products within the most frequently-recognized CD8-target pool ([ORF28/ORF36/ORF37], which was recognized by 7 out of 14 individuals) have been previously investigated for immunogenicity. Of these three proteins, ORF28 is a likely immunogenic candidate, as it has been classified as an envelope glycoprotein based on positional and structural similarities to EBV BDLF3 [47], which encodes the EBV glycoprotein gp150. BDLF3 is a known target of the EBV-specific CD4 response [41], and although KSHV ORF28 is its positional equivalent, its gene product shares no amino acid sequence similarity to EBV gp150. In the search for immunodominant virus-specific epitopes it seems logical to focus on viral gene products with unique sequences, as epitopes derived from such proteins are the most likely to be biologically relevant in terms of effective host control of viral infection. KSHV ORF36 encodes a serine protein kinase [48] and ORF37 a modified DNA exonuclease involved in host mRNA shut off [49]. Both these genes' products share considerable amino acid sequence similarity with the products of their respective EBV homologues, BGLF4 and BGLF5. BGLF5 has not been identified as a target of the EBV-specific T-cell response and although CD8 responses to BGLF4 have been detected in one individual [43], it is not a major T-cell target. Thus these two KSHV gene products seem less likely immunogenic candidates, although this remains to be investigated.

Interestingly, [ORF8/ORF49/ORF61] and [ORF59/ORF65/K4.1] were also common CD4 targets, recognized by 6 of 14 and 4 of 14 individuals, respectively. As previously mentioned, very little is known of the CD4 response against KSHV. However it is not surprising that it is directed against the same gene products that elicit the strongest CD8 responses. In the T-cell response against HCMV, 53% of the most recognized ORFs are common to both the CD8 and CD4 response [46]. Similarly, the major EBV CD8 targets BZLF1 and BMLF1 (immediate-early lytic) and EBNA1, EBNA2, EBNA3A, EBNA3B, EBNA3C and LMP2 (latent) also elicit CD4 responses [41], [42]. Further studies will elucidate the most antigenic KSHV gene products from within each pool, and will identify new CD8 and CD4 epitopes.

The present study has enabled a far broader investigation into the targets of both the CD8 and CD4 KSHV-specific T-cell responses than has been previously undertaken. We found, unexpectedly, that these responses were skewed towards early and late lytic gene products. This knowledge will be important to future immunological investigations into KSHV and may eventually lead to the development of better immunotherapies for KSHV-related diseases, and potentially even a vaccine against KSHV.

## Materials and Methods

### Ethics Statement

All KSHV-infected and non-infected study participants were recruited from Chelsea and Westminster Hospital, London, UK, and provided written, informed consent. The study protocols were approved by Riverside Research Ethics Committee.

### Study Participants

Five groups of study participants were studied. 1) HIV-negative, KSHV-seronegative, healthy individuals (n = 3); 2) HIV-positive, KSHV-seronegative individuals (n = 4); 3) HIV-positive, KSHV-seropositive individuals with a history of KSHV-related disease (KS; KS and MCD; or PEL) but in remission at time of venesection (n = 11); 4) HIV-positive, KSHV-seropositive individuals with no history of KSHV-related disease (asymptomatic carrier; n = 1); and 5) HIV-negative, KSHV-seropositive individuals with active KSHV-related disease (KS), in regression at time of venesection (n = 2).

All study participants were male apart from one HIV-positive, KSHV-seronegative female. Study participants' age on date of venesection ranged from 30 to 74 years. All HIV-positive individuals were on HAART and had an HIV viral load of <50 copies per mL. CD4 counts ranged from 171 to 785 cells/mm^3^. Characteristics of each group of study participants can be seen in Table 1.

### Construction of KSHV-gene-encoding lentiviral vectors and lentivirus production

The lentiviral vector pSIN-MCS was derived from the green fluorescence protein (GFP)-encoding vector pCSGW (a kind gift from Professor Adrian Thrasher, Institute of Child Health, University College London). Packaging plasmids p8.91 and pMD.G were a kind gift from Professor Didier Trono, Ecole Polytechnique Fédérale de Lausanne, Switzerland). KSHV ORFs were cloned from cDNA or genomic DNA from the BC3 PEL cell line or from previously constructed vectors into pSIN-MCS as previously described [39], [50]. Primers used in cloning of each gene are available on request. Vesicular stomatitis virus-G envelope-pseudotyped lentiviral virions were produced also as previously described [39], [50]. Briefly, 293T cells were cotransfected for 5 hours with 2 µg of pSIN-MCS, 1.5 µg of p8.91 and 1.5 µg of pMD.G using the standard FuGENE (Roche, East Sussex, UK) protocol. Transfected 293Ts were then cultured for a further 48 hours in fresh media (DMEM [Gibco, Invitrogen, Paisley, UK] supplemented with 10% FCS and Penicillin-Streptomycin [both Sigma, Poole, UK]), after which the virion-containing supernatant was harvested, filtered (0.45 µm) and stored at −80°C for later use.

### Generation of monocyte-derived dendritic cells (moDCs) from peripheral blood

Peripheral blood mononuclear cells (PBMCs) and plasma were isolated from whole blood using Histopaque-1077 Hybri-Max gradient separation (Sigma). 1 ml aliquots of plasma were stored at −80°C for later use in serological testing. PBMCs were separated into CD14+ and CD14- fractions using a standard immunodepletion protocol utilizing magnetic beads labeled with a monoclonal antibody directed against CD14 (Miltenyi Biotech, Bergisch Gladbach, Germany). CD14- monocytes were stored for later use at −80°C in a freezer mix comprised of 50% RPMI 1640, 40% fetal calf serum (FCS) and 10% dimethyl sulfoxide (DMSO) (all Sigma).

CD14+ monocytes were plated at 3×10^5^ cells per well on 24-well plates (Cellstar, Greiner Bio-one, Gloucestershire, UK) and cultured in RPMI with Hepes modification and supplemented with L-Glutamine, Penicillin-streptomycin and 5% Human AB Serum (Lot number 027K0432) (all Sigma). Differentiation to moDCs was induced by addition of 70 ng/ml interleukin 4 (IL-4) and 70 ng/ml granulocyte/macrophage colony-stimulating factor (GMCSF) (both R and D systems, Oxford, UK). Cells were fed with fresh media and cytokines every second day until maturation on day 8 (see below).

### Lentiviral transduction of moDCs

On day 4 in culture, moDCs were transduced by incubation with the appropriate volume of each lentiviral preparation (typically between 150 µl and 600 µl, calculated according to the multiplicity of infection [MOI] of each preparation). Lentiviral preparations were thawed on ice for 30 minutes then warmed to 37°C in a water bath directly prior to use. Lentivirus was then added directly to the cells in 24-well plates. On day 8 in culture (day 4 post-transduction) moDCs were matured by stimulation with a “cytokine cocktail” made up from 5 ng/ml tumor necrosis factor α (TNFα), 5 ng/ml interleukin 1β (IL-1β), 150 ng/ml interleukin 6 (IL-6) (all R and D systems) and 1 µg/ml prostaglandin E2 (PGE2; Sigma). On day 10 in culture (day 6 post-transduction), moDCs were harvested for use in further experiments. Expression of constructs was confirmed by reverse transcriptase PCR (RT-PCR). GFP expression by pCSGW-infected moDCs was assessed by fluorescent microscopy and flow cytometry performed on a FACSCalibur (BD Biosciences, Oxford, UK). To determine the MOI of each lentiviral preparation, quantitative real time PCR (qPCR) was performed for the lentiviral packaging signal using the glyceraldehyde-3-phosphate dehydrogenase (GAPDH) gene as the reference signal, as previously described [50].

### Transduction of moDCs with an adenovirus encoding the CMV gene phosphoprotein 65

moDCs transduced to express the immunodominant CMV gene phosphoprotein 65 (CMVpp65) were used as a positive control. Unfortunately, despite extensive efforts, we were unable to clone CMVpp65 into our lentiviral vector. We therefore used an adenovirus encoding CMVpp65 (Adpp65) to transduce moDCs. Adpp65 was a kind gift from Dr Magnus Essand of Uppsala University and we used the previously published protocol for transduction [51]. Briefly, on day 8 in culture, immature moDCs were harvested by aspiration, spun at 1400 rpm for 8 minutes, resuspended in 250 µl media and counted. moDCs were transduced with Adpp65 (MOI = 300) for 2 hours at 37°C. moDCs were than plated on 24-well plates and maturation stimulus was added as above.

### Immunophenotyping of moDCs

moDCs transduced with 300 µl pCSGW (MOI = 5.6) were analyzed for expression of cell surface markers by multi-parameter flow cytometry. moDCs were stained with monoclonal antibodies against HLA-A,B,C conjugated to APC (mouse anti-human, IgG1κ; BD Biosciences) and CD80 conjugated to PE (mouse anti-human, IgG1κ; BD Biosciences) or their appropriate isotype controls, at a final antibody dilution of 1 in 20, for 30 minutes on ice, protected from light. Flow cytometry was performed on a FACSCalibur (Becton Dickinson) and analyzed with CELLQuest software (Becton Dickinson). 10 000 events were collected for each sample. moDCs were gated on the live-cell population according to the expected forward scatter and side scatter. Isotype controls were used to set gates for positive staining for each of the antibodies.

### Serology for KSHV

An in-house developed MIX-MAP ELISA against was used to detect KSHV seropositivity as previously described [7] but with an improved, modified peptide containing two copies each of a lytic and a latent epitope (RSHLGFWQEGWSGQVYQDWLGRMNCSYENM derived from K8.1, and QPGPSREYRYVLRTSPPHRPGVRMRRV derived from ORF 73, respectively).

### HLA Typing

Genomic DNA was extracted from a minimum of 5×10^6^ PBMCs using the standard Nucleon® BACC2 kit (Tepnel Life Sciences, Manchester, UK) protocol. HLA typing was performed by the Department of Histocompatibility and Immunogenetics, Clinical Immunology Laboratory, Hammersmith Hospital, London. Low resolution typing was performed using PCR-sequence specific primers (PCR-SSP). High resolution typing was achieved using reference strand conformational analysis (RSCA).

### T cell Purification

T cells were isolated from thawed cryogenically-preserved CD14- PBMCs using a standard protocol for immunodepletion using magnetic beads labeled with monoclonal antibodies directed against CD14, CD16, CD19, CD36, CD56, CD123 and Glycophorin A (Miltenyi Biotech).

### T-cell Proliferation Assays

Carboxy Fluorescein Succinimidyl Ester (CFSE; Molecular Probes, Invitrogen, Paisley, UK) was reconstituted in DMSO to make a 9 mM stock solution. CFSE stock solution was diluted to a 10 µM working solution in PBS. Purified T cells were resuspended in 100 µl PBS and 100 µl 10 µM CFSE solution per 10^6^ cells for 5 minutes at room temperature and then blocked with 200 µl FCS per 10^6^ cells for 15 minutes at room temperature.

CFSE-stained T cells were then plated for culture in proliferation reactions with autologous transduced moDCs. Cell mixtures were cultured in round-bottomed 96-well plates (Cellstar) in a total volume of 200 µl T cell media (RPMI 1640 with Hepes modification supplemented with L-Glutamine, Penicillin-Streptomycin and 10% Human AB Serum, Lot number 027K0432). T cells were cultured at 100, 000 cells per well with 2, 500 autologous moDCs (transduced or non-transduced) per well. In addition, for each experiment the following control wells were set up: 1) T cells only 2) T cells+non-transduced moDCs+5 µg/ml phytohemagglutinin (PHA; Sigma) 3) T cells+moDCs transduced with Adpp65.

After 6 days of culture, cells were harvested, stained with a monoclonal antibody against CD8 conjugated to PeCy5 (mouse, anti-human, IgG1κ; BD Biosciences) or the appropriate isotype control at a final antibody dilution of 1 in 20, for 30 minutes on ice, protected from light. Flow cytometry was performed on a FACSCalibur (Becton Dickinson) and analyzed with CELLQuest software (Becton Dickinson). The maximum possible events (typically between 10 000 and 30 000) were collected for each sample. T cells were gated on the live-lymphocyte population according to the expected forward scatter and side scatter, and then on either CD8-high or CD8-low (CD4) T cell populations (see Figure 3a).

### Statistical Analysis

Statistics were performed on Microsoft Office Excel 2003 software. For T cell responses to transduced moDCs, a positive response was designated as a response that fitted three criteria of significance above the background response to moDCs transduced with the empty lentiviral vector: 1) p<0.05; unpaired student T test 2) response >3.5 standard deviations above background and 3) response >10% above background. A borderline response was designated as p<0.05 and response >3 standard deviations and >9 per cent above background.
